# Bioinformatics and metabolic flux analysis highlight a new mechanism involved in lactate oxidation in *Clostridium tyrobutyricum*

**DOI:** 10.1007/s10123-022-00316-y

**Published:** 2023-01-07

**Authors:** Edouard Munier, Hélène Licandro, Eric Beuvier, Rémy Cachon

**Affiliations:** 1 Univ. Bourgogne Franche-Comté, L’Institut Agro Dijon, PAM UMR A 02.102, 21000 Dijon, France; 2 INRAE, URTAL, 39800 Poligny, France

**Keywords:** EtfAB phylogenetic analysis, Lactate oxidation, Butyric acid fermentation, *Clostridium tyrobutyricum*, Metabolic flux analysis

## Abstract

Climate change and environmental issues compel us to find alternatives to the production of molecules of interest from petrochemistry. This study aims at understanding the production of butyrate, hydrogen, and CO_2_ from the oxidation of lactate with acetate in *Clostridium tyrobutyricum* and thus proposes an alternative carbon source to glucose. This specie is known to produce more butyrate than the other butyrate-producing clostridia species due to a lack of solvent genesis phase. The recent discoveries on flavin-based electron bifurcation and confurcation mechanism as a mode of energy conservation led us to suggest a new metabolic scheme for the formation of butyrate from lactate-acetate co-metabolism. While searching for genes encoding for EtfAB complexes and neighboring genes in the genome of *C. tyrobutyricum*, we identified a cluster of genes involved in butyrate formation and another cluster involved in lactate oxidation homologous to *Acetobacterium woodii*. A phylogenetic approach encompassing other butyrate-producing and/or lactate-oxidizing species based on EtfAB complexes confirmed these results. A metabolic scheme on the production of butyrate, hydrogen, and CO_2_ from the lactate-acetate co-metabolism in *C. tyrobutyricum* was constructed and then confirmed with data of steady-state continuous culture. This in silico metabolic carbon flux analysis model showed the coherence of the scheme from the carbon recovery, the cofactor ratio, and the ATP yield. This study improves our understanding of the lactate oxidation metabolic pathways and the role of acetate and intracellular redox balance, and paves the way for the production of molecules of interest as butyrate and hydrogen with *C. tyrobutyricum*.

**Supplementary information:**

The online version contains supplementary material available at 10.1007/s10123-022-00316-y.

## Introduction

Many saccharolytic species of the genus *Clostridium* are known to produce CO_2_, butyric acid, and H_2_ as the main metabolic end products of butyric acid fermentation during the acidogenesis phase (Lee et al. 2008). Over the past decades, butyric acid fermentation has attracted great research and development (R and D) interest as it offers an alternative for the production of (i) butyric acid, a platform molecule widely used in food, cosmetic, and oil industries and mainly produced through petrochemical means, and (ii) hydrogen, a potential environmentally friendly energy carrier for substitution of fossil fuels and mainly produced from water electrolysis (Zeng and Zhang 2010; Cai et al. 2010; Dwidar et al. 2012; Bao et al. 2020).

*C. tyrobutyricum* produces higher amounts of butyric acid and hydrogen as the main fermentation products from carbohydrates than other species like *Clostridium acetobutylicum* or *Clostridium butyricum* (Cummins and Johnson 1971; Jang et al. 2013, 2014). As a consequence, this bacteria has been extensively explored by metabolic engineering and in fermentation for its potential to produce butyric acid and hydrogen from sugars and lignocellulosic biomass hydrolysates containing glucose and xylose (Liu et al. 2006; Jo et al. 2010; Jiang et al. 2013; Du et al. 2015; Yu et al. 2015; Fu et al. 2017; Suo et al. 2018a, b, 2019; Li et al. 2020). Among species usable in an industry, *C. tyrobutyricum* seems to be the best candidate in particular due to its sole acidogenesis phase (Jaros et al. 2013). *C. tyrobutyricum* is also able to metabolize lactate to butyrate and hydrogen (Cheng et al. 2013).

Lactic acid production by fermentation has attracted a lot of interest as it offers an alternative to the environmental pollution caused by the petrochemical industry (Abdel-Rahman et al. 2013). Lactic acid has a wide range of applications such as in food, beverages, cosmetics, pharmaceuticals, and chemical industry, and hence its demand is continuously increasing (Jantasee et al. 2017). From an industrial perspective, the use of lactate could be an attractive alternative and cost-effective to the use of carbohydrate to produce a molecule of interest, especially since it has been shown that lactate co-metabolism with acetate could stimulate butyrate and bio-hydrogen productions (Matsumoto and Nishimura 2007). But the mechanism of lactate oxidation in *C. tyrobutyricum* has not yet been identified and the functioning of the metabolic pathway has not been clarified. Moreover, studies on lactate oxidation metabolism could bring a solution to the late blowing defect in the cheese industry caused by contamination by *C. tyrobutyricum* (Klijn et al. 1995; D’incecco et al. 2018).

The first suggestion of lactate oxidation mechanism was proposed in *C. acetobutylicum*. Lactate oxidation to pyruvate would be catalyzed with iNAD^+^ lactate dehydrogenase, but the genes involved have not yet been identified (Cheng et al. 2013). The discovery of flavin-based electron bifurcation (FBEB) complexes has improved our understanding of energy conservation mechanisms in strict anaerobic bacteria and archaea (Thauer et al. 2008; Wang et al. 2010; Buckel and Thauer 2018a). This conservation of energy results in a net exergonic reaction with minimal negative free energy change involving an electron transfer between exergonic and endergonic reactions. FBEBs complexes are divided into four distinct families, including that of the electron transfer flavoprotein (Etfs) complex. This complex consists of two subunits encoded by the *etfA* and *etfb* genes. It was first described in *Clostridium kluyveri* (Bcd/EtffAB) as its genes are clustered with a butyryl-CoA dehydrogenase gene (Li et al. 2008). This key reaction in the production of butyrate involves the exergonic reduction of crotonyl-CoA to butyryl-CoA with NADH and the endergonic reduction of ferredoxins with NADH, the equation (Eq. 1) of which is as follows:

1 $$Crotonyl-CoA+2 NADH+2 {Fd}_{ox}\to butyryl-CoA+2 {NAD}^{+}+2 {Fd}_{red}$$

Genes encoding BCD/EtfAB are clustered with HBD ones (3-hydroxybutyryl-CoA dehydrogenase) also found in other *Clostridium* genomes (Bennett and Rudolph 1995; Lee et al. 2016). It is common to find genes coding for the Etfs complex in the same genetic locus as a CoA dehydrogenase gene in bacterial genomes. This is the case for *A. woodii* with a lactate dehydrogenase (LDH) gene. This LDH/EtfAB complex explains the biochemical reaction of lactate oxidation (Weghoff et al. 2015). It involves the exergonic reaction of the ferredoxin oxidation reduced with NAD^+^ coupled with the endergonic reaction of lactate reduction to pyruvate with NAD^+^ giving the following equation (Eq. 2):

2 $$Lactate+2 {NAD}^{+}+2 {Fd}_{red}\to pyruvate+2 NADH+2 {Fd}_{ox}$$

This FBEB complex leads to decrease in the ΔG°' of the oxidation of lactate from + 25 to + 6 kJ mol^−1^ making the reaction energetically more favorable. Genes encoding the LDH/EtfAB complex are grouped with those encoding a lactate permease, a lactate racemase, and a transcriptional regulator forming a lactate oxidation operon. Orthologous operons have been identified in other *Firmicutes* species, some of which belong to the *Clostridium* genus such as *Clostridium botulinum* and *Clostridiuml jundhalii*. From phylogenetic and structural analyses of different EtfA and EtfB subunits belonging to the domain of bacteria and archaea, five Etf groups (G1–G5) have been distinguished. The G2 group is mostly represented by the *Firmicutes* and divided into two sub-groups. The G2A subgroup includes the Etfs involved in butyrate synthesis, and the G2B subgroup comprises those in lactate oxidation (Garcia Costas et al. 2017).

The aim of this study was to investigate lactate-acetate co-metabolism in *C. tyrobutyricum* using the LDH/EtfAB complex. Because *C. tyrobutyricum* is phylogenetically close to *C. ljungdhalli*, a comparison with *A. woodii* and *C. kluyveri* could provide answers to the metabolic mechanisms of lactate oxidation involved. The investigation was made in three complementary steps: (i) *in silico* analysis of putative lactate oxidation clusters, (ii) phylogenetic analysis, and (iii) metabolic flux analysis (MFA) including intracellular redox balance and carbon recovery, with data gathered from a steady-state continuous culture of *C. tyrobutyricum* using lactate and acetate as carbon substrates.

## Methods

### Identification of a predicted EtfAB and neighboring genes involved in lactate oxidation from *Clostridium tyrobutyricum* CIRM BIA 2237

Protein sequence research studies from predicted Etf genes were performed on *C. tyrobutyricum* CIRM BIA 2237 (NCBI RefSeq: NZ_CP038158.1). The *etfA* (locus_tag: AWO_RS04415) and *etfB* (AWO_RS0441) genes of *A. woodii* DSM 1030 (NC_016894) (Weghoff et al. 2015) were used as queries. Searches, data analyses, and visualizations were performed with NCBI blast. The *etf* sequences from *C. ljungdahlii* DSM13528 were also used as queries.

### Phylogenetic analysis of Etf complex proteins from the selected bacterial species

EtfA and EtfB protein sequences were searched in the genome sequence of the following species: *A. woodii* DSM1030 (RefSeq: NC_016894), *C. kluyveri* DSM555 (RefSeq: NC_009706.1), *Clostridium acetobutylicum* ATCC 824 (RefSeq: NC_003030.1), *Clostridium butyricum* KNU-L09 (RefSeq: NZ_CP013252.1), *Clostridium beijerenckii* DSM791 (RefSeq: NZ_CP073653.1), *C.tyrobutyricum* CIRM BIA 2237 (RefSeq: NZ_CP038158.1), and *Eubacterium limosum* (RefSeq: NZ_CP019962.1).

A custom EtfA and EtfB protein database of the above listed species was prepared using the NCBI database by searching all electron transfer flavoprotein A or B genes from the genome sequences.

The selected EtfA and EtfB protein sequences forming an Etf complex in each genome were compilated by addition of both sequences giving Etf complex protein sequences.

Etf complexes were subjected to phylogenetic analysis, and protein sets were aligned using ClustalW with default parameters (cost matrix: ID, gap open cost: 8, gap extend cost: 0.1). A phylogenetic tree was built using Tree Builder with the following parameters: genetic distance model: Jukes-Cantor (Jukes and Cantor 1969), tree build method: UPGMA (Michener and Sokal 1957), resampling method: bootstrap, number of replicates: 1000, support threshold: 30% by using the Geneious v2022.0.2 software.

### Bacteria and medium

*C. tyrobutyricum* CIRM BIA 2237 was obtained from National Institute of Agronomic Research and Environment collection. The strain was stored at – 80 °C in cryotubes. For pre-culture, 1 colony of *C. tyrobutyricum* on RCM (reinforced clostridial medium; Biokar, France) Petri dishes was used to inoculate 100 mL of an RCM-modified (glucose was replaced by sodium lactate) medium at 37 °C until obtaining OD_600nm_: 1.4–1.8 measured by a spectrometer (PRIM; Secomam, France). A medium with 14 g.L^−1^ sodium lactate (Sigma-Aldrich, France), yeast extract 3 g.L^−1^ (VWR, France), and 10 g.L^−1^ meat extract (VWR, France), 3 g.L^−1^ sodium acetate (Rectapur, France), 5 g.L^−1^ NaCl (Chem-LAB, Belgium), 0.05 g.L^−1^ cysteine hydrochloride (Sigma-Aldrich, France) was used in the pre-culture and continuous culture studies. Media for the pre-culture were autoclaved at 120 °C for 15 min and filter-sterilized (0.2 µm) for continuous cultures (Millipore, Merck, France).

### Continuous culture setup

Continuous culture was carried out in a 2-L bioreactor with 750 mL working volume (New Brunswick, Scientific, Discovery 100, USA). The bioreactor was autoclaved with distilled water at 120 °C for 15 min. After cooling, water was replaced by the filter-sterilized medium with lactate and acetate. The culture was controlled at a temperature of 37 °C, and a pH of 5.8. The stirring rate was controlled at 150 rpm, which was high enough to keep a homogenous medium without leading to significant foaming. Oxidoreduction potential (*E*_h_) was measured with redox probe (Pt4805-DPAS-SC K8S; Mettler Toledo, France). The initial batch culture was started with 700 mL of a medium inoculated with 50 mL pre-culture, and switched to the continuous culture at the end of the growth phase. During fermentation, nitrogen was sparged into the headspace of the bioreactor at a flow rate of 10 mL.min^−1^ to maintain the anaerobic state of the bioreactor. The feed medium flow rate was controlled by a peristaltic pump (MINIPULS 3; Gilson, France). A total volume of 750 mL was maintained using a balance (Lynx; Mettler Toledo, France) coupled with a monitoring reactor unit (MRU) (New Brunswick, USA).

### Analytical methods

Short-chain fatty acids were analyzed by HPLC with ion exclusion (HPLC system; Diomex (Thermo Fisher), Photodiode Array Detector Ultimate 3000 DAD, and a 300 × 7.8 mm Aminex HPX-87H column with a guard column). HPLC conditions for determination of organic acids were: column temperature of 30 °C; flow rate, 0.6 mL.min^−1^; injected volume, 20 µL; mobile phase, 9 mM sulfuric acid in water.

#### *In silico* model construction and metabolic flux

**Fig. 1:**
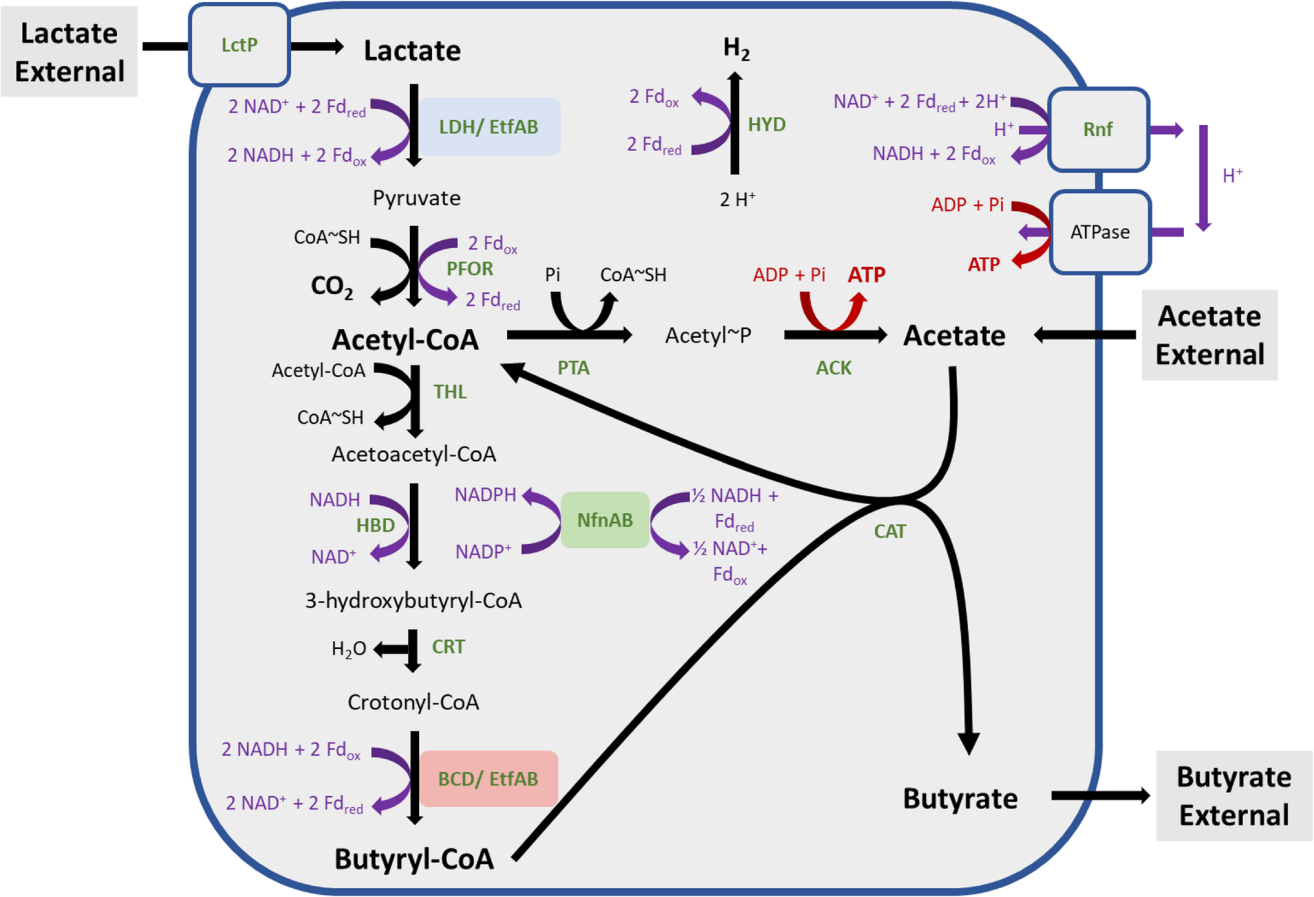
Proposed scheme for the metabolic pathways involving lactate and acetate conversion in* C. tyrobutyricum*. LctP, lactate permease; LDH, lactate dehydrogenase; PFOR, pyruvate ferredoxine:oxidoreductase; PTA, phosphotransacetylase; ACK, acetate kinase; THL, thiolase; HBD, 3-hydroxybutyryl-CoA dehydrogenase; CRT, crotonase; BCD, butyryl-CoA dehydrogenase; CAT, CoA-transferase; HYD, hydrogenase

The anaerobic lactate oxidation metabolic network for the *C. tyrobutyricum* CIRM BIA 2237 strain was developed for MFA from the identification of EtfAB complex in *C. tyrobutyricum* CIRM BIA 2237 and phylogenetic analysis steps and completed with pathway information collected from other studies reported in the literature and the KEGG database (Lee et al. 2016; Kanehisa et al. 2017). The metabolic network of *C. tyrobutyricum* CIRM BIA 2237 was mathematically constructed with four key reactions (Table 1), gathering 11 reactions and seven intracellular metabolites from *C. tyrobutyricum* metabolism (Fig. 1). The lactate and acetate uptakes and the production of butyrate, which were measured during the steady-state phase of the continuous culture, were used as constraints in MFA. The steady-state phase was identified from the stability of the cell density determined by the OD_600nm_ method with stable lactate and acetate consumption values and redox potential value (*E*_h_). The model construction and the in silico analysis were carried out from the reactions in Table 1 using a previous method (Tsai and Lee 1988; Orth et al. 2010) and applied on https://smart-biotech.online/research/mfa/clostridium.tyrobutyricum.lactate.

**Table 1:** Reactions used in the metabolic model of *C. tyrobutyricum* lactate oxidation metabolism

Numbers	Reactions
R1	CH_2_O → C_2/3_HO_1/3_CoA_1/3_ + C_1/3_O_2/3_
R2	CH_3/2_O_1/2_CoA_1/2_ → CH_3/2_O
R3	2 CH_3/2_O_1/2_CoA_1/2_ → CH_7/4_O_1/4_CoA_1/4_
R4	C_2/3_H_7/6_O_1/6_CoA_1/6_ + C_1/3_H_1/2_O_1/3_ → C_2/3_H_7/6_O_1/6_ + C_1/3_H_1/2_O_1/6_CoA_1/6_

The carbon equations were constructed by dividing the atom numbers by the C number of the substrate

From the metabolic steady state for intracellular metabolites, the metabolic fluxes ($v$) are constrained by the stoichiometry matrix (*S*) giving the following equation:

3 $$Sv=0$$

Metabolic carbon fluxes were calculated with measured rates (*r*) of lactate and acetate consumption, butyrate production, estimating CO_2_ production rate from lactate-acetate metabolic scheme (Fig. 1), and lactate consumption rate, giving the matrix system in Eq. (4), where *x*_*i*_ are intracellular flow rates*.*

Considering the steady-state continuous culture, the intracellular flow rates of acetyl-CoA and butyryl-CoA are assumed as equal to 0.

4 $$\left|\begin{array}{c}\begin{array}{c}{r}_{lactate}\\ {r}_{CO2}\\ {r}_{acetylCoA}\\ {r}_{acetate}\\ {r}_{butyrylCoA}\\ {r}_{butyrate}\end{array}\end{array}\right|=\left|\begin{array}{c}\begin{array}{cccc}-1& 0& 0& 0\\ 1/3& 0& 0& 0\\ 2/3& -1& -1& 1/3\\ 0& 1& 0& -1/3\\ 0& 0& 1& -2/3\\ 0& 0& 0& 2/3\end{array}\end{array}\right|\times \left|\begin{array}{c}x1\\ x2\\ x3\\ x4\end{array}\right|$$

An important application of MFA includes determining ATP yields and parameters of redox homeostasis (cofactor redox balance and metabolites redox balance, both being involved in cellular redox balance).

ATP yield (*Y*_ATP,_ g.mol^−1^) is the ratio of biomass (g DW.L^−1^) produced from ATP (mol.L^−1^). For cofactor recovery, NADH ratio (Eq. 5) and Fd ratio (Eq. 6) were calculated separately and compiled together, giving Eq. 7. All these ratios were calculated on the basis of intracellular flux:

5 $$\frac{\sum NAD+}{\sum NADH}\times 100$$

6 $$\frac{\sum Fdox}{\sum Fdred}\times 100$$

7 $$\frac{\sum Fdox +\sum NAD+}{\sum Fdred+\sum NADH}\times 100$$

Redox balance was calculated from the oxidation state of substrates and products (lactate, 0; acetate, 0; carbon dioxide, + 2; hydrogen, − 1; and butyrate, − 2) (Riondet et al. 2000). The sum of the oxidation state of products should be equal to the sum of the oxidation state of substrates. Considering that the sum of the oxidation state of substrates equals 0, the following equation was used for redox balance (Eq. 8):

8 $$\frac{\sum {r}_{\mathrm{product} }\times \mathrm{product} \;\mathrm{oxidation}\; \mathrm{state}}{{r}_{\mathrm{substrates}}}$$

Dividing by *r*_substrates_ allows to compare conditions with different substrates flow rates.

## Results and discussion

### Identification of genes involved in lactate oxidation

The discovery of complex FBEBs has led to a better understanding of the metabolic function of strict anaerobes and archaea (Buckel and Thauer 2018a), and to understand some metabolic activities like the ability to metabolize lactate with acetate or butyrate synthesis from some *Clostridium* species (Li et al. 2008; Detman et al. 2019).

**Fig. 2:**
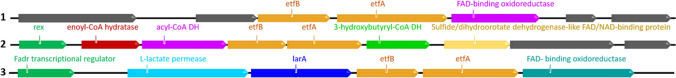
Chromosome fragments (NZ_CP038158.1) of* C. tyrobutyricum CIRM BIA 2237 *containing EtfAB and FAD-binding oxidoreductase genes. EtfB (1: EZN00_RS10860, 2: EZN00_RS10795, 3: EZN00_RS08615), EtfA (1: EZN00_RS10865, 2: EZN00_RS10800, 3: EZN00_RS08620), FAD-binding oxidoreductase (1:: EZN00_RS10870; 3: EZN00_RS08625)

We studied the genome of *C. tyrobutyricum* CIRM BIA 2237 to show the presence of EtfAB complexes. *C. tyrobutyricum* CIRM BIA 2237 has been isolated from silage and has already been used as a lactate oxidizer in different works (Roux and Bergere 1977). Its complete genome has been fully sequenced and annotated (Munier et al. 2019). Analysis of the chromosome (NZ_CP038158.1) of *C. tyrobutyricum* CIRMBIA 2237 identified three clusters of genes coding for EtfAB complexes (Fig. 2).

In fragment 1, genes encoding the EtfAB complex are next to a FAD-binding oxidoreductase. The EtfAB complex involved in fragment 2 is in a gene cluster involving an enoyl-CoA hydratase (also identified as crotonase), an acyl-CoA dehydrogenase, a 3-hydroxybutyryl-CoA dehydrogenase, and a FAD/NAD-binding protein. The predicted acyl-CoA dehydrogenase protein presents 100% identity with the butyryl-CoA dehydrogenase of *C. tyrobutyricum* KCTC 5387 and can thus be identified as a butyryl-CoA dehydrogenase. Therefore, the cluster of genes identified in fragment 2 is most likely involved in the production of the butyryl-CoA characteristic of butyrate-producing *Clostridium*.

In fragment 3, the EtfAB complex is included between the coding genes for a transcriptional regulator, a L-lactate permease, a lactate racemase, and a FAD-binding oxidoreductase. This gene cluster was also found in *C. ljungdahlii* DSM 13528, a species phylogenetically close to *C. tyrobutyricum* (Collins et al. 1994; Weghoff et al. 2015).

**Fig. 3:**
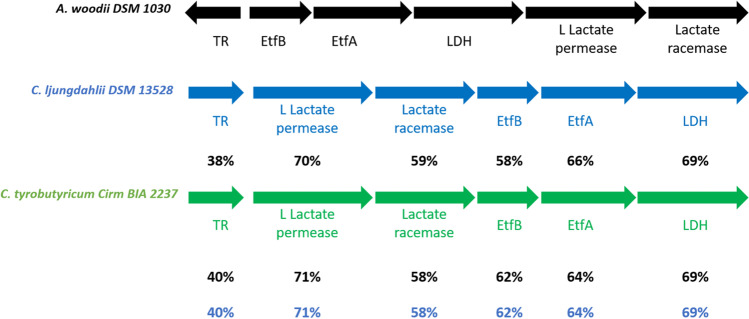
Putative lactate oxidation cluster of* C. tyrobutyricum CIRM BIA 2237. *Its identity percentage is compared with those from* C. ljungdahlii DSM 13,528 et A. woodii DSM 1030*. TR, transcriptional regulator (EZN00_RS08600, CLJU_RS10615, AWO_RS0440); L-lactate permease (EZN00_RS08605, CLJU_RS10610, and AWO_RS04425); lactate racemase (EZN00_RS08610, CLJU_RS10605, and AWO_RS04430); EtfB (EZN00_RS08615, CLJU_RS10600, and AWO_RS04410); EtfA (EZN00_RS08620, CLJU_RS10595, and AWO_RS04415); LDH, lactate dehydrogenase (EZN00_RS08625, CLJU_RS10590, and AWO_RS04420)

The protein sequences found in the lactate oxidation cluster present at least 38% of identity and 95% of query cover between *C. tyrobutyricum* CIRM BIA 2237, *C. ljungdahlii* DSM 13528, and *A. woodii* DSM 1030 (Fig. 3).

According to these results, *C.tyrobutyricum* would have the same mechanism of lactate oxidation as *A. woodii*, i.e., involving an EtfAB complex.

In order to support our proposal, a phylogenetic comparison was made based on EtfAB protein sequences of different species that can oxidize lactate and produce butyrate (*E. limosum* ATCC 8486, *C. butyricum* KNU-L09, *C. beijerenckii* DSM 791, and *C. acetobutylicum* ATCC 824), a reference species for the oxidation of lactate (*A. woodii*), and a reference species for the production of butyrate (*C. kluyveri*). This phylogenetic analysis was restricted to bacteria able to uptake lactate in the presence of acetate to produce butyrate. A search for EtfAB complexes was previously carried out by BLASTp in these different selected species. In the genomes of the selected species, at least two EtfAB complexes have been identified.

**Fig. 4:**
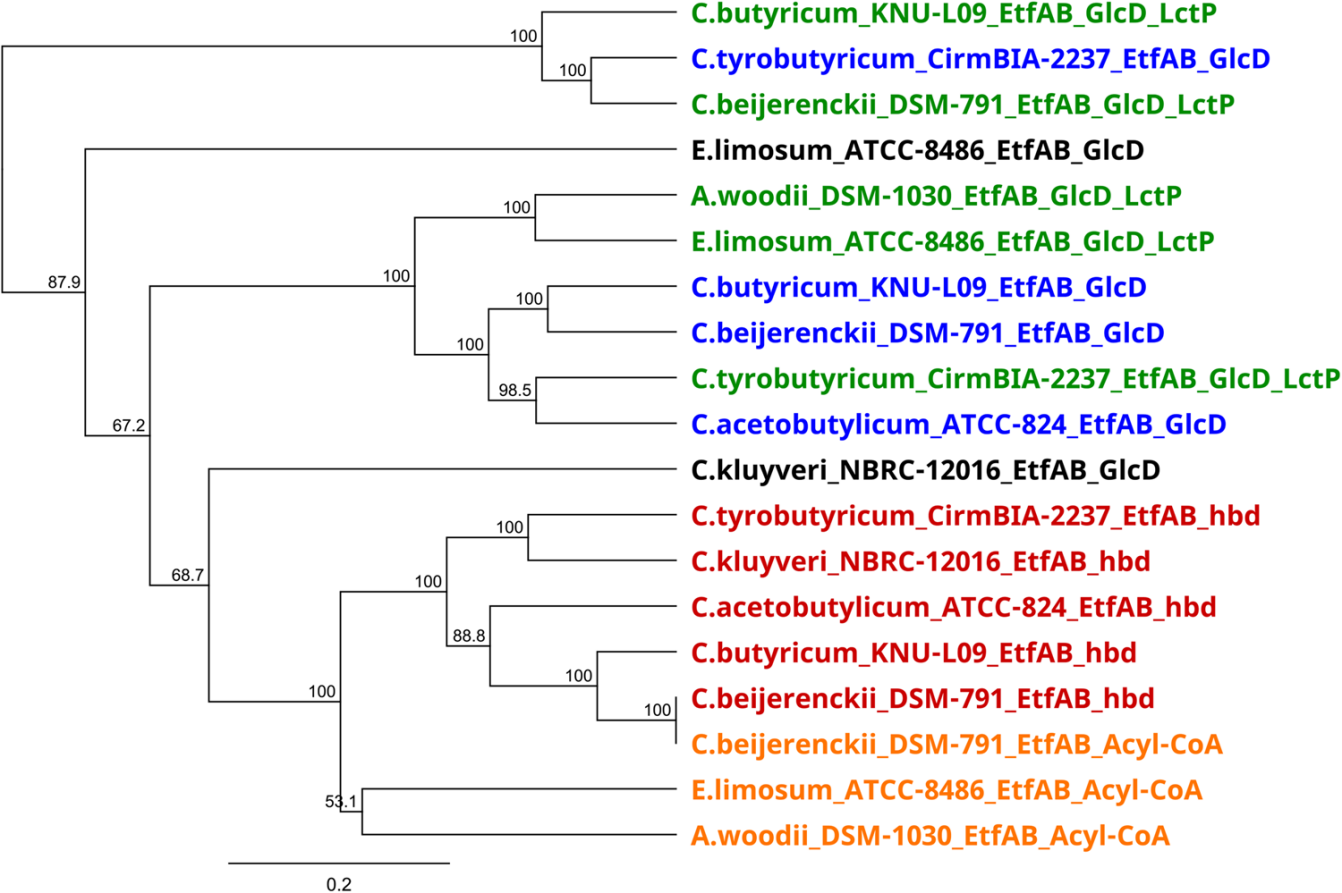
Phylogenetic analysis of Etf complex protein sequences between different species. Orange, EtfAB coupled with acyl-CoA DH; red, EtfAB coupled with Hbd; green, EtfAB coupled FAD-binding oxidoreductase/LDH and lactate permease; blue, EtfAB-coupled FAD-binding oxidoreductase; black, out of any phylogenetic group

As shown in Fig. 4, the EtfAB complexes encoded by the *etfA* and *etfB* genes associated with the *hbd* form a distinct group with 100% bootstrap support.

As already observed in other studies, the latter is related to EtfAB complexes associated with an acyl-CoA dehydrogenase gene (Detman et al. 2019). Concerning EtfAB complexes associated with a gene coding for GlcD or GlcD/LctP, two distinct additional groups are observable. The first forms a cluster involving the EtfAB complex associated with GclD/LctP in *A. woodii* and *C. tyrobutyricum* with 100% bootstrap support, and the second groups together with the EtfABGlcD_LctP complexes of *C. butyricum* KNU-L09 and *C. beijerenckii* DSM 791 as well as that associated with GlcD in *C. tyrobutyricum* CIRM BIA 2237.

Our results are in agreement with those of Garcia Costas et al. (2017) and Detman et al. (2019). The two G2 subgroups of EtfAB are found in this phylogenetics analysis. EtfAB protein sequences encoded by *etfA* and *etfB* associated with *hbd* or acyl-CoA DH belonging to subgroup G2A are involved in butyrate production. Those with the gene encoding for GlcD and/or LldP_GlcD including *A. woodii* formed subgroup G2B involved in lactate oxidation. The results of this phylogenetic analysis correspond to the ones obtained by comparisons of protein sequences with *A. woodii* and *C. ljungdahlii*.

### Metabolic scheme with enzyme machinery of lactate and acetate transformation to butyrate

An update of the metabolic scheme involving the oxidation of lactate in *C. tyrobutyricum* could be proposed (Fig. 1)*.* This scheme was built from previous studies on butyric acid fermentation from *C. tyrobutyricum* (Lee et al. 2016; Kanehisa et al. 2017), the database KEGG, and knowledge of flavin-based electron confurcation and regulation of energy conversion mechanisms (Buckel and Thauer 2018a).

We constructed a model of central carbon metabolism in *C. tyrobutyricum* involving the conversion of lactate to pyruvate via a stable cytoplasmic complex formed of LDH with electron-transferring flavoprotein (EtfAB) using flavin-based electron confurcation. Unlike in glucose where two ATPs are produced during glycolysis, this scheme with the LDH/EtfAB complex proposes the acetate branch as the only metabolic pathway capable of producing ATP with the F_0_F_1_ ATPase and covering the cell’s energy needs. Moreover, the reaction of the electron confurcation involving oxidation of reduced ferredoxins will change the intracellular redox balance.

Electron confurcation is the reverse reaction of bifurcation coupling an exergonic reduction of NAD^+^ with reduced ferredoxin with an endergonic reduction of NAD^+^ and lactate in our case. As explained in Buckel and Thauer (2018b), two different electron donors, one from reduced ferredoxin (low potential donor) and one from lactate (high potential donor), act together to reduce the bifurcating NAD^+^.

#### *In silico* model construction and verification

To support the results obtained from the bioinformatics analyses, a MFA model was built. The metabolic flux network of *C. tyrobutyricum* CIRM BIA 2237 was constructed from the scheme in Fig. 1 without including Rnf complex and NfnAB because a previous work has shown that the genes coding for these complexes are poorly expressed during butyric fermentation (Lee et al. 2016). Therefore, a first MFA with only Etfs complexes and hydrogenases was carried out. Lactate and acetate utilization and production of butyrate, three of the main substrates and products of fermentation, were followed during the continuous culture (Table 2). These continuous data were used as constraints in the stochiometric matrix (Eq. 4). Batch data from the literature were also used as constraints in the MFA model as second verification (Bryant and Burkey 1956). Carbon recovery and butyrate/acetate flux ratio were used as criteria to evaluate the agreement between stochiometric matrix and experiments.

**Table 2:** Results of continuous culture and batch fermentation in liquid medium with lactate and acetate as carbons sources

Fermentation	Continuous culture	Batch culture
	(This study)	(Bryant and Burkey 1956)
Acetate consumed (mol.mol^−1^ lactate)	0.30	0.37
Butyrate produced (mol.mol^−1^ lactate)	0.65	0.63
CO_2_ produced (mol.mol^−1^ lactate) ^a^	1.00	0.97
Carbon recovery (%)	100	92
B/A ratio (mol.mol^−1^)^b^	2.17	1.7
Y_ATP_(g DW.mol^−1^ATP)	8.82	-

Acetate, butyrate, and CO_2_ values are expressed from 1 mol of lactate consumed

^a^CO_2_ produced in continuous culture was determined from the amount of lactate consumed, according to Fig. 1

^b^Ratio of butyrate produced/acetate consumed

As can be seen in Table 2, data from fermentations with two different strains and culture processes lead to similar acetate/lactate, butyrate/lactate, and CO_2_/lactate ratios and consequently a butyrate/acetate ratio around 2, and a carbon recovery around 100%. Consequently, the following general equation of lactate-acetate co-metabolism in *C. tyrobutyricum* could be proposed:

9 $$3 lactate+1 acetate\to 3 {CO}_{2}+2 butyrate$$

**Fig. 5:**
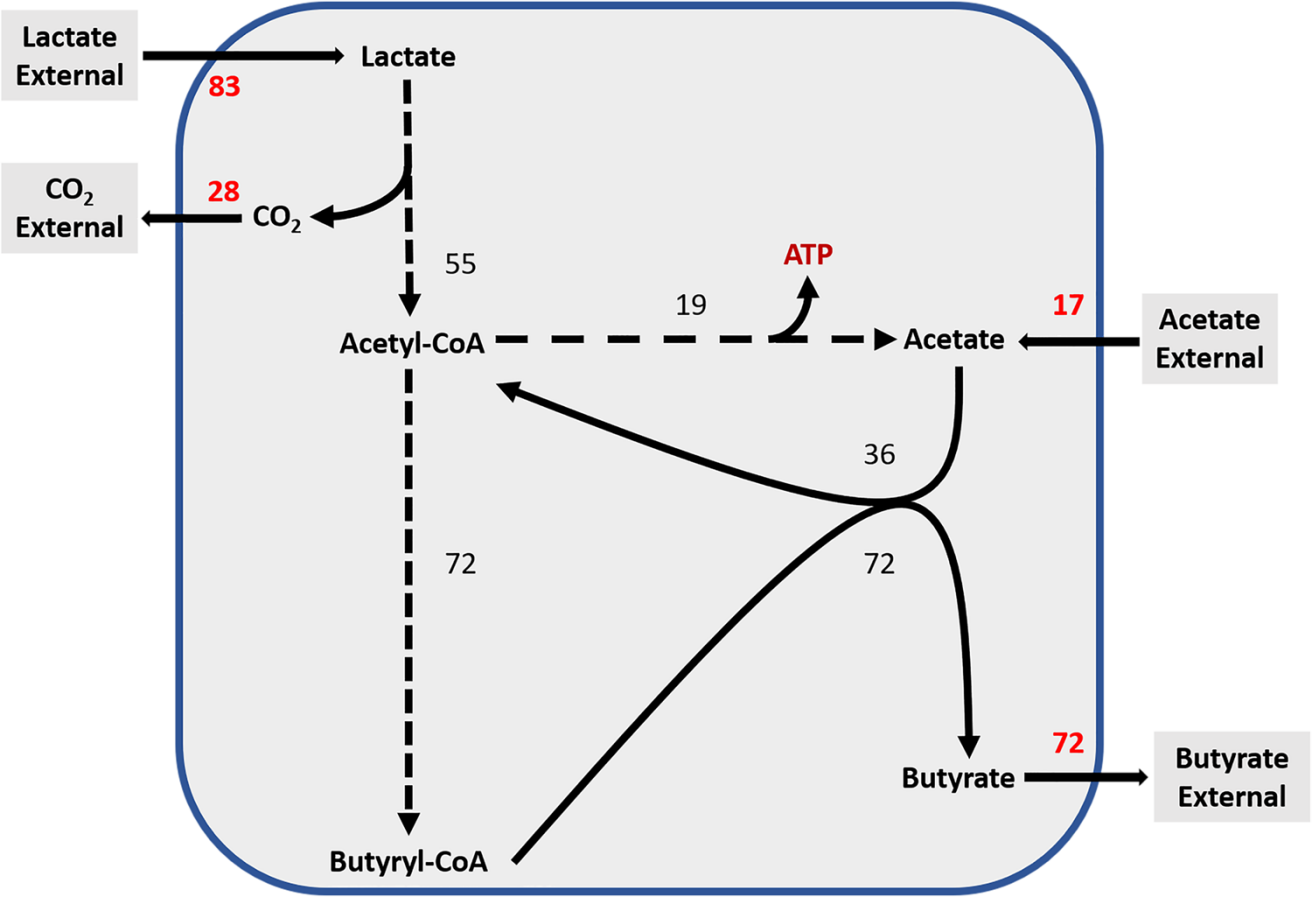
Scheme of the metabolic flux distribution involving lactate and acetate conversion by *C. tyrobutyricum*. Using an MFA model with 100 mM–C of substrates (83 mM–C from lactate and 17 mM–C from acetate). All values are expressed in % of total carbon. Hatched arrows represent multiple metabolic reactions

The MFA model allows to quantify intracellular metabolic fluxes (all expressed in % mM–C) (Fig. 5); starting from 83 mM–C of lactate and 17 mM–C of acetate external for a total of 100 mM–C, carbon flux distribution from lactate is majorly flowed until acetyl-CoA (55 mM–C) node with a production of CO_2_ (28 mM–C). Flux from acetyl-CoA node was bifurcated into acetic acid formation (19 mM–C) and butyric acid synthesis (72 mM–C) via butyryl-CoA node. The assimilation of 17 mM–C of external acetate with the 19 mM–C of acetate formed justifies the 72 mM–C of the butyrate branch.

Thus, butyrate production is possible by addition of external acetate carbons and re-assimilation of acetate carbons formed in metabolic pathways. Moreover, we could observe that the consumed external acetate is essential in intra-cellular homeostasis. Indeed, by removing it from MFA, we found that the NADH ratio had fallen from 98 to 87%. This result was also observed in *Staphylococcus aureus* metabolism (Marshall et al. 2016).

Figure 5 is in agreement with Eq. 9; the sum of carbon fluxes giving acetyl-CoA at the origin of butyrate (72 mM–C) production respected a ratio of 3 coming from lactate (55 mM–C) and 1 from acetate (17 mM–C). Two acetyl-CoA are required for the production of one butyryl-CoA. To confirm the consistency of these observations with a necessary recovery of both CoA–SH and NADH, a statement from this stoichiometric balance was performed from intracellular metabolic rates calculated by MFA (Table 3), and the following routes were considered according to Fig. 1:

- For NAD^+^/NADH ratio (Eqs. 5 and 7): LDH/EtfAB, HbBD, and BCD/EtfAB routes
- For Fd_ox_/Fd_red_ ratio (Eqs. 6 and 7): LDH/EtfAB, PFOR, BCD/EtfAB, and HYD routes

**Table 3:** Results of NADH, ferredoxin, and intracellular redox ratios and oxidation state balance with and without hydrogen

Ratio and balance	*C. tyrobutyricum* CIRM BIA 2237	
	Without hydrogen	With hydrogen
NADH ratio, Eq. (5) (%)	98	98
Ferredoxin ratio, Eq. (6) (%)	61	100
Cofactor recovery, Eq. (7) (%)	75	99
Oxidation state balance, Eq. (8)	0.53	0.03

A NADH ratio of 98% is obtained. However, without the HYD route, a 61% recovery could be calculated for ferredoxin ratio and causing an excess of 2.34 mM.h^−1^ of reduced ferredoxin. This result can be explained due to the absence of the hydrogen-produced value from the hydrogenase during fermentation (Fig. 1).

By respecting the reaction stoichiometry involving hydrogenase, 1.14 mM.h^−1^ of hydrogen would be generated considering the results obtained with the calculation of intracellular fluxes (Table 3). In order to confirm this result, a second approach was carried out using oxidation state balance (Eq. 8). Considering intracellular fluxes, 0.03 of oxidation state balance was obtained instead of 0.53 without hydrogen value. The first value is closer to neutrality, meaning that hydrogen production should be necessary to maintain redox homeostasis. Moreover, the HYD route improved the ferredoxin ratio to 100% and cofactor recovery to 99% (Table 3). These results are close to full balance; it suggests a low expression of genes coding for the Rnf and NfnAB complexes in consumption of lactate and acetate. Furthermore, with the agreement of these MFA results with consistency between the flow distribution and intracellular redox ratio close to 100% of recovery, the involvement of LDH/EtfAB in the lactate oxidation mechanism from *C. tyrobutyricum* would be metabolically possible.

Concerning ATP synthesis, an ATP yield at 0.35 mol.mol^−1^ of substrates was calculated. This result is similar to those obtained with propionate fermentation from *Clostridium homopropionicum* and *Veillonella parvula* from lactate uptake (Seeliger et al. 2002). Besides, an 8.8 g DW. mol^−1^ ATP yield is in agreement with the 9 g DW. mol^−1^ ATP estimated from *C. kluyveri* with ethanol and acetate as substrates and similar to or higher than *C. butyricum* (10.1 gDW. mol^−1^ ATP) and *E. coli* (7.9 g DW. mol^−1^ ATP) with glucose as substrate, further confirming our suggestion (Thauer et al. 1968; Crabbendam et al. 1985; Riondet et al. 2000). Thus, despite the constraints of continuous data and stoichiometry of metabolic pathways, we obtained a consistency between flow distribution, an intracellular redox recovery close to 100%, and a positive ATP generation value close to lactate oxidation in other bacteria. These results support the consistency of this new scheme proposed with LDH/EtfAB.

## Conclusion

Bioinformatics allowed us to identify a gene cluster coding for the LDH/EtfAB complex that could be involved in lactate oxidation metabolism from *C. tyrobutyricum.* The metabolic flux analysis involving the LDH/EtfAB complex has confirmed our assumptions about the lactate oxidation mechanism in *C. tyrobutyricum*. In addition, the lactate/acetate ratio of 3:1 provides an explanation of how acetate is used as a co-substrate for the production of two butyrate molecules. The understanding of this metabolism could allow for the development of new strategies against this bacterium known to be involved in late blowing defect of cheese, or even exploit lactate and acetate as new resources to produce hydrogen or butyrate as molecules of interest.

## Supplementary information

Below is the link to the electronic supplementary material.

Supplementary file1 (DOCX 16 KB)

## Data Availability

All the data generated or analyzed during this study are included in this published article. The datasets used and/or analyzed during the current study are available from the corresponding author upon reasonable request and on the following website: https://smart-biotech.online/research/mfa/clostridium.tyrobutyricum.lactate

## Abbreviations

*Etf*: Electron transfer flavoprotein
*FAD*: Flavin adenine dinucleotide
*GlcD*: Domain of FAD-dependent lactate dehydrogenase
*FAD/FMN*: containing dehydrogenase
*HPLC*: High-performance liquid chromatography
*LDH*: Lactate dehydrogenase
*LldP*: Lactate permease
*OD*: optical density
*R&D*: Research and development
*iNAD*^*+*^: Lactate dehydrogenase: independent NAD^+^ lactate dehydrogenase
*FBEB*: Flavin-based electron bifurcation
*Etf*: Electron transfer flavoprotein
*Bcd*: Butyryl-CoA dehydrogenase
*HBD*: 3-Hydroxybutyryl-CoA dehydrogenase
*MFA*: Metabolic flux analysis
*RCM*: Reinforced clostridial medium
*KEGG*: Kyoto Encyclopedia of Genes and Genomes
*DH*: Dehydrogenase
*THL*: Thiolase
*PTA*: Phophotransacetylase
*CRT*: Crotonase
*ACK*: Acetate kinase
*PFOR*: Pyruvate ferredoxin:oxidoreductase
*Rnf*: Ferredoxin:NAD reductase
*NfnAB*: NADH-dependent ferredoxin:NADP reductase
*Hyd*: Hydrogenase
*DW*: Dry weight
