# Supplementary material for: Bioinformatics and metabolic flux analysis highlight a new mechanism involved in lactate oxidation in Clostridium tyrobutyricum
Source: Int Microbiol. 2023 Jan 7;26(3):501–11. doi: 10.1007/s10123-022-00316-y (PMC10397141; doi:10.1007/s10123-022-00316-y)
Supplement: Supplementary file 1 — Supplementary file1 (DOCX 16 KB) [file 10123_2022_316_MOESM1_ESM.docx]

# Supplementary material

**Detailed genetic information used for phylogenetic analysis**:

For EtfA:*A._woodii*_DSM-1030_EtfA (AWO_RS04415), *A_.woodii*_DSM-1030_EtfA (AWO_RS08105), *C._butyricum*_KNU-L09_EtfA (ATN24_08885), *C._butyricum*_KNU-L09_EtfA (ATN24_03030), *C._butyricum*_KNU-L09_EtfA (ATN24_03165), *C._tyrobutyricum*_CIRMBIA-2237_EtfA (EZN00_RS08620), *C._tyrobutyricum*_CIRMBIA-2237_EtfA (EZN00_RS10800), *C._tyrobutyricum*_CIRMBIA-2237_EtfA (EZN00_RS10865), *C._kluyveri*_NBRC-12016_EtfA (CKR_RS02265), *C._kluyveri*_NBRC-12016_EtfA (CKR_RS16650), *C._beijerenckii*_DSM-791_EtfA (KEC93_RS01655), *C._beijerenckii*_DSM-791_EtfA (KEC93_RS01720), *C._beijerenckii*_DSM-791_EtfA (KEC93_RS10410), *C._beijerenckii*_DSM-791_EtfA (KEC93_RS14700), *C._acetobutylicum*_ATCC-824_EtfA (CA_RS13965), *E.limosum*_ATCC-8486_EtfA (B2M23_RS01165), *E.limosum*_ATCC-8486_EtfA (B2M23_RS12010), *E.limosum*_ATCC-8486_EtfA (B2M23_RS16675), *C._ ljungdahlii*_DSM-13528_EtfA (CLJU_RS10595). For EtfB: *A.woodii*_DSM-1030_EtfB (AWO_RS04410), *A.woodii*_DSM-1030_EtfB (AWO_RS08100), C._butyricum_KNU-L09_EtfB (ATN24_08880), *C._butyricum*_KNU-L09_EtfB (ATN24_03025), *C._butyricum*_KNU-L09_EtfB (ATN24_03160), *C._tyrobutyricum*_CIRMBIA-2237_EtfB (EZN00_RS08615), *C._tyrobutyricum*_CIRMBIA-2237_EtfB (EZN00_RS10795), *C._tyrobutyricum*_CIRMBIA-2237_EtfB (EZN00_RS10860), *C._kluyveri*_NBRC-12016_EtfB (CKR_RS02260), *C._kluyveri*_NBRC-12016_EtfB (CKR_RS16645), *C._beijerenckii*_DSM-791_EtfB (KEC93_RS01650), *C._beijerenckii*_DSM-791_EtfB (KEC93_RS01715), *C._beijerenckii*_DSM-791_EtfB (KEC93_RS10405), *C._beijerenckii*_DSM-791_EtfB (KEC93_14705), *C._acetobutylicum*_ATCC-824_EtfB (CA_RS13035), *C._acetobutylicum*_ATCC-824_EtfB (CA_RS13970), *E._limosum*_ATCC-8486_EtfB_ (B2M23_RS01170), *E._limosum*_ATCC-8486_EtfB (B2M23_RS12005), *E._limosum*_ATCC-8486_EtfB (B2M23_RS16670), *C._ljungdahlii*_DSM-13528_EtfB (CLJU_RS10600).

List of renamedEtf complex proteins with the neighboring genes with which they were found:*A._woodii*_DSM-1030_EtfAB_GlcD_LctP, *A._woodii*_DSM-1030_EtfAB_Acyl-CoA, *C._butyricum*_KNU-L09_EtfAB_GlcD_LctP, *C._butyricum*_KNU-L09_EtfAB_GlcD, *C._butyricum*_KNU-L09_EtfAB_Hbd, *C._tyrobutyricum*_CIRMBIA-2237_EtfAB_GlcD_LctP,*C._tyrobutyricum*_CIRMBIA-2237_EtfAB_Hbd, *C._tyrobutyricum*_CIRMBIA-2237_EtfAB_GlcD, *C._kluyveri*_NBRC-12016_EtfAB_Hbd, *C._kluyveri*_NBRC-12016_EtfAB_GlcD, *C._beijerenckii*_DSM-791_EtfAB_GlcD, *C._beijerenckii*_DSM-791_EtfAB_Hbd, *C._beijerenckii*_DSM-791_EtfAB_Acyl-CoA, *C._beijerenckii*_DSM-791_EtfAB_GlcD_LctP, *C._acetobutylicum*_ATCC-824_EtfAB_GlcD, *C._acetobutylicum*_ATCC-824_EtfAB_Hbd, *E._limosum*_ATCC-8486_EtfAB_Acyl-CoA, *E._limosum*_ATCC-8486_EtfAB_GlcD, *E._limosum*_ATCC-8486_EtfAB_GlcD_LctP, *C._ljungdahlii _*DSM-13528_EtfAB_GlcD_LctP.
